# Effectiveness and Safety of Irreversible Electroporation When Used for the Ablation of Stage 3 Pancreatic Adenocarcinoma: Initial Results from the DIRECT Registry Study

**DOI:** 10.3390/cancers16233894

**Published:** 2024-11-21

**Authors:** Robert C. G. Martin, Rebekah Ruth White, Malcolm M. Bilimoria, Michael D. Kluger, David A. Iannitti, Patricio M. Polanco, Chet W. Hammil, Sean P. Cleary, Robert Evans Heithaus, Theodore Welling, Carlos H. F. Chan

**Affiliations:** 1 Division of Surgical Oncology, Department of Surgery, University of Louisville, Louisville, KY 40202, USA; sean.cleary@uhn.ca (S.P.C.);; 2 San Diego Moores Cancer Center, University of California, La Jolla, CA 92037, USA; 3 Northwest Community Healthcare, Arlington Heights, IL 60005, USA; 4 Columbia University Herbert Irving Comprehensive Cancer Center, New York, NY 10032, USA; 5 Atrium Health Carolinas Medical Center, Charlotte, NC 28203, USA; 6 University of Texas Southwestern Medical Center, Dallas, TX 75390, USA; 7 Siteman Cancer Center, Washington University School of Medicine in St. Louis, St. Louis, MO 63110, USA; 8 Mayo Clinic, Rochester, MN 55905, USA; 9 University Health Network, University of Toronto, Toronto, ON M5G 2C4, Canada; 10 Department of Radiology, College of Medicine, University of South Florida, Tampa, FL 33606, USA; 11 NYU Langone Health, New York, NY 10016, USA; 12 UC San Diego Health, San Diego, CA 92037, USA; 13 University of Iowa Hospitals and Clinics, Iowa City, IA 52242, USA

**Keywords:** irreversible electroporation, IRE, pancreatic ductal adenocarcinoma, PDAC, ablation, NanoKnife

## Abstract

Background/Objectives: Overall survival for patients with Stage 3 pancreatic ductal adenocarcinoma (PDAC) remains limited, with a median survival of 12 to 15 months. Irreversible electroporation (IRE) is a local tumor ablation method that induces cancerous cell death by disrupting cell membrane homeostasis. The DIRECT Registry study was designed to assess the effectiveness and safety of IRE when combined with standard of care (SOC) treatment for Stage 3 PDAC versus SOC alone in a real-world setting after at least 3 months of induction chemotherapy; Methods: Patients with Stage 3 PDAC treated with IRE plus SOC or SOC alone were prospectively enrolled in a multicenter registry study. Enrollment required 3 months of active multi-agent chemotherapy with no progression before enrollment. Endpoints were 30- and 90-day mortality and adverse events (AEs). Results: Eighty-seven IRE and 27 SOC subjects were enrolled in the registry. Mean ages were 64.0 ± 8.4 and 66.4 ± 9.9 years, and mean anterior/posterior tumor diameters were 2.2 ± 0.7 cm and 3.2 ± 1.3 for the IRE and SOC groups respectively (*p* = 0.0066). All IRE procedures were performed using an open approach. The 90-day all-cause mortality was 5/83 (6.0%) and 2/27 (7.4%) for the IRE and SOC groups, respectively. Two subjects in the IRE group died from treatment-related complications, and one patient in the SOC group died due to chemotherapy-related complications. Conclusions: Initial results from the DIRECT registry study indicate the use of IRE for curative intent tumor ablation in combination with induction chemotherapy has equivalent morbidity and mortality rates when compared to standard-of-care chemotherapy alone.

## Simple Summary

The DIRECT Registry study is a prospective trial of irreversible electroporation (IRE) using the NanoKnife System for the treatment of patients with Stage 3 pancreatic adenocarcinoma (PDAC). The data from this United States Food and Drug Administration Investigational Device Exemption study aims to assess the safety and effectiveness of IRE in a real-world setting in the treatment of Stage 3 PDAC after induction chemotherapy when combined with standard of care (SOC) compared to SOC alone.

## 1. Introduction

The incidence of pancreatic cancer has risen consistently, with an estimated 66,000 individuals to be diagnosed with pancreatic cancer in 2024, and approximately 52,000 patients with pancreatic cancer will die from the disease [1]. Pancreatic cancer is currently the ninth most frequently diagnosed cancer and the third leading cause of cancer-related death in the United States [2]. The median survival time for patients with locally advanced pancreatic Stage 3 cancer (LAPC) undergoing pancreatic resection following neoadjuvant therapy is 20 months [3]. Patients with Stage 3 pancreatic ductal adenocarcinoma (PDAC) represent 39.2% of the nonmetastatic patient population and have a 5-year survival of 10.8% [3]. The probability of survival is inversely proportional to tumor size and the number of positive lymph nodes [4].

The current standard of care (SOC) for Stage 3 PDAC includes systemic chemotherapy with either FOLFIRINOX (combination chemotherapy using 5-fluorouracil (5-FU), leucovorin (folinic acid), irinotecan, and oxaliplatin) or a combination of albumin-bound (nab) paclitaxel and gemcitabine therapy. FOLFIRINOX has demonstrated improved overall survival (OS) to 11.1 months for FOLFIRINOX versus 6.4 months for gemcitabine alone in metastatic pancreatic cancer, but at the cost of greater toxicity [5]. A modified form of FOLFIRINOX (without the bolus 5-FU and with a reduced dose of irinotecan) has been shown to have an acceptable safety profile while maintaining comparable efficacy of FOLFIRINOX in metastatic pancreatic cancer [6]. The National Cooperative Cancer Network (NCCN) currently recommends either modified FOLFIRINOX (mFOLFIRINOX) or nab-paclitaxel and gemcitabine as a preferred regimen for Stage 3 pancreatic cancer patients with a good performance status (ECOG 0–1) [7].

Although radiation therapy is frequently utilized in the U.S. for patients with Stage 3 pancreatic cancer, there currently exists limited Level 1 evidence demonstrating the benefit of radiation. One of the pivotal studies for radiation therapy in this population was the LAP07 prospective randomized trial [8]. In this trial, patients received induction chemotherapy with gemcitabine, with or without erlotinib, and were then randomized to receive chemoradiation therapy or continue chemotherapy. There was no difference in the primary endpoint of OS, although there was decreased local tumor progression in the chemoradiation arm. One major limitation of this trial is that the induction chemotherapy used was not consistent with the current standard of using multi-agent combination chemotherapy and more effective systemic induction regimens, such as FOLFIRINOX or nab-paclitaxel and gemcitabine.

The poor outcomes among patients with advanced pancreatic cancer have led to the pursuit of new treatment options. Irreversible electroporation, or IRE (NanoKnife System, AngioDynamics, Inc., Latham, NY, USA), is a non-thermal-based method for local ablation which causes increased permeabilization of the cell membrane through the exposure of the cell to electric pulses [9]. Electrodes are placed in a pattern that enables the tumor to be encircled by the electrical field produced, with electric pulses irreversibly permeating the membranes, resulting in cell death. Initial prospective and retrospective studies to date suggest that IRE treatment may increase the median OS of LAPC and may lead to better preservation of vessels, nerves, and extracellular matrix within or close to the ablated area, compared to thermal ablation techniques [9,10,11,12,13,14,15].

The DIRECT Registry study [NCT NCT03899649] is a U.S. Food and Drug Administration (FDA) approved Investigational Device Exemption (IDE) study designed to prospectively investigate the safety and efficacy of IRE treatment combined with SOC compared to SOC alone in patients with Stage 3 PDAC, with the goal of facilitating the enrollment of a broad patient population using a real-world registry study approach.

## 2. Materials and Methods

This DIRECT registry is a multicenter, observational, non-randomized study that enrolled patients with Stage 3 PDAC who received SOC alone or IRE in addition to SOC. The study received approval from the Western Institutional Review Board (IRB approval 2019-0965), and all subjects provided written informed consent prior to enrollment. All subjects were required to have undergone at least 3 months of SOC multi-agent chemotherapy without progression prior to enrollment in the registry. The DIRECT Registry study enrolled both control and IRE patients from sites where patients are routinely treated with ablation using the NanoKnife System. Additional control patients were enrolled from sites that do not offer IRE treatment. The primary objectives for the DIRECT Registry study are to test the hypothesis that IRE with the NanoKnife System improves survival in subjects with Stage 3 PDAC and to assess the safety of IRE compared to the control SOC cohort.

### 2.1. Key Eligibility Criteria

Subjects with cytologically or pathologically confirmed pancreatic adenocarcinoma that were unresectable and who met the study’s inclusion/exclusion criteria were eligible for enrollment. The study was designed to have broad inclusion/exclusion criteria enabling the evaluation of real-world evidence regarding the safety and effectiveness of IRE in subjects who had received a wide variety of prior treatments (chemotherapy, chemoradiation, or other procedures). All subjects were required to have a confirmed diagnosis of Stage 3 PDAC according to the American Joint Committee on Cancer (AJCC) staging criteria and National Comprehensive Cancer Network (NCCN) guidelines and have undergone at least 3 months of SOC multi-agent chemotherapy without progression, based on NCCN Guidelines, prior to enrollment in the registry [7,16]. Additional inclusion criteria included an age of 18 years or older, an axial and anterior to posterior tumor dimension of ≤3.5 cm after standard of care, and an American Society of Anesthesiologists (ASA) classification of the physical health status of 1, 2, 3, or 4. Exclusion criteria included site participation in another interventional trial for pancreatic cancer during the study data collection period, pregnant or lactating patients or those of child-bearing potential not willing to use birth control from screening to 6 months after the last dose of chemotherapy, those unable to tolerate general anesthesia with full skeletal muscle blockade and patients with an implanted cardiac pacemaker, defibrillator, electronic device(s), or implanted device(s) with metal parts in the thoracic cavity at the time of IRE treatment.

### 2.2. Primary Study Endpoints

The primary endpoint for this initial report of results from the DIRECT study was mortality for any reason between enrollment in the registry and at both 30 and 90 days. There were two co-primary safety endpoints for the study. The first was the development of a new onset CTCAE v5.0 Grade 3 or higher adverse event (AE) between enrollment in the registry and day 90. The second was the development of a CTCAE v5.0 Grade 4 or higher chemotherapy-related AE between enrollment and day 90 for the SOC group or the development of a Grade 4 or higher treatment-related AE for the IRE group during the 90-day time period after IRE treatment.

### 2.3. Irreversible Electroporation Procedure

The use and delivery of intraoperative IRE with the NanoKnife System has been previously described [14,17,18,19,20]. The surgically placed IRE electrodes were inserted under ultrasound guidance with the number of electrodes necessary to achieve a complete electroporation zone with an adequate margin of at least 5 mm determined by the target lesion size based on both axial/anterior–posterior and cranial/caudal dimensions. The electrodes were typically placed in a caudal to cranial fashion after appropriate dissection, except when used for margin accentuation. When used for margin accentuation, the IRE energy was delivered before complete dissection/transection since soft tissue is still in place, enabling IRE electrode insertion. The position of the electrodes in relation to the tumor and/or vessels was assessed in real time and adjusted to maximize the treatment effect. The delivery of IRE was considered successful based on intraoperative ultrasonography and real-time assessment of resistance change in the ablation zone [21].

### 2.4. Statistical Analysis

Statistical analysis was performed by the study sponsor, with the present analysis being limited to the study’s primary endpoints. The statistical analyses conducted for quantitative variables included frequency counts and percentages, means, standard deviations (SD), and minimums and maximums of each parameter. Frequencies and percentages summarized categorical variables. Unless explicitly stated, percentages utilized a denominator corresponding to the number of unique subjects or lesions that contributed to the endpoint. Statistical analyses were run using SAS Software version 9.4 (SAS Institute Inc., Cary, NC, USA).

## 3. Results

### 3.1. Patient Population

A total of 114 subjects were consented and enrolled in the registry between May 2019 and May 2023. This included 87 subjects in the IRE arm and 27 subjects in the SOC arm. Baseline demographics and clinical characteristics for each arm were similar (Table 1) except for a higher percentage of CA19-9-expressing patients in the SOC arm. Supplemental Table S1 lists the prior medical histories of subjects enrolled in the registry, which were similar in both groups. Overall, the two subject populations were similar except for a greater race diversity in the SOC arm. Vascular, gastrointestinal, and metabolic/nutritional disorders were the most common under medical conditions for both groups. Supplemental Table S2 reports the pre- and post-enrollment surgical history for subjects in the IRE and SOC groups, which were also similar.

### 3.2. Tumor Characteristics

The majority of tumors in the study population were located in either the head of the pancreas only (51.8%) or only in the body or neck (42.1%), with the former being slightly more prevalent for the IRE group (54.0 vs. 44.4%) and the latter for the SOC group (48.1% vs. 40.2%) (Table 2). The mean anterior/posterior tumor diameter was larger for the SOC group compared to the IRE group (3.2 ± 1.3 vs. 2.2 ± 0.7 cm; *p* = 0.0066), as was the cranial/caudal tumor diameter with a mean anterior/posterior diameter of compared to for the IRE group (3.0 ± 0.8 vs. 2.4 ± 0.8 cm; *p* = 0.0052).

### 3.3. Neoadjuvant and Radiation Treatment

All subjects underwent induction chemotherapy, with 76.3% of the total study population receiving FOLFIRINOX alone or combined with other chemotherapeutic agents (Table 2). A higher percentage of patients in the SOC group were treated with FOLFI-RINOX as standalone therapy compared to the IRE group (66.7% vs. 49.4%, respectively, *p* = 0.1166). The SOC group also had a lower mean number of chemotherapy cycles before enrollment than the IRE group (4.6 ± 2.8 vs. 5.7 ± 5.0 cycles, respectively, *p* = 0.1645). While 37 (42.5%) of subjects in the IRE group had prior radiation therapy, no subjects in the SOC group were reported to have had prior radiation therapy.

### 3.4. IRE Treatment Data

Table 3 summarizes data associated with IRE procedures. All procedures were performed using an open approach, with 50 (57.5%) for in-situ tumor ablation and 37 (42.5%) with the intent of margin accentuation in combination with resection. The mean IRE delivery time was 55.7 ± 54.5 min per procedure, with 2 to 4 probes (range 2 to 6) most commonly used and a mean of 1031.1 ± 882.1 pulses delivered per procedure. The mean total procedure time was 338.0 ± 174.2 min. When reported, all subjects had an IRE pulse length between 70 to 90 µs, indicating an adequate range required for complete treatment. Thirty-five (40.2%) subjects required a mean of 2.0 ± 1.1 probe pullbacks to achieve adequate tumor coverage for treatment.

A total of 70 (80.5%) subjects underwent additional adjunctive surgical procedures at the same time as IRE treatment (Table 3). The most common concomitant procedures were cholecystectomy (33.3%), Whipple procedure (28.7%), J-tube placement (28.7%), lymphadenectomy (14.9%), and either distal or subtotal pancreatectomy (11.5%).

### 3.5. Safety Overview

The 30-day all-cause mortality following enrollment in the DIRECT Registry was similar for both study arms, with 2 (2.3%) deaths in the IRE arm and 1 (3.7%) death in the SOC arm. The 90-day mortality was also similar, with 5 (6.0%) and 2 (7.4%) deaths in the IRE and SOC groups, respectively.

**Figure 1:**
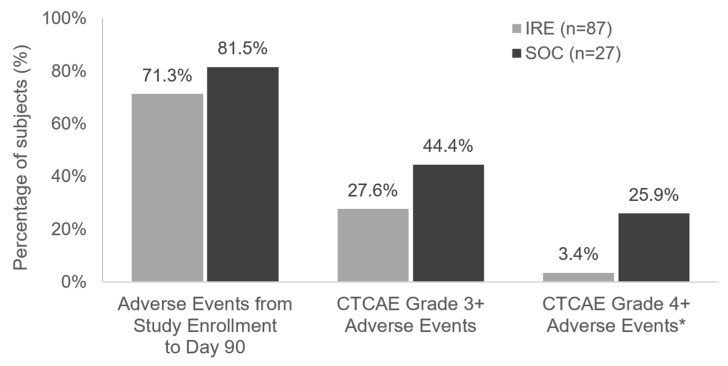
Adverse events. * For Grade 4+ events associated with IRE treatment for the IRE study arm and chemotherapy for the SOC study arm.

A total of 62 (71.3%) subjects in the IRE group and 22 (81.5%) in the SOC group experienced AEs during the 90-day time period following enrollment in the registry (Figure 1). Twenty-four (27.6%) subjects in the IRE group and 12 (44.4%) in the SOC group experienced a Grade 3 or higher AE during the same time period (Table 4).

Three (3.4%) subjects in the IRE group experienced a total of four Grade 4 or higher IRE treatment-related AEs during the 90-day time period following treatment (Table 5). All four AEs were classified as directly related to IRE treatment. One subject experienced two separate events that were reported on the same date (cardiac arrest and septic shock) with complete recovery and resolution. The remaining two subjects who developed IRE treatment-related AEs (abdominal hemorrhage and vascular pseudoaneurysm) died as a result of these complications. Three subjects in the IRE treatment group developed postoperative pancreatic fistulas, with all recovering without long-term effects. All three were considered less than CTCAE v5.0 Grade 3 adverse events.

Seven (25.9%) subjects in the SOC group experienced a total of 12 chemotherapy-related Grade 4 or higher AEs (Table 5). Four of these AEs were classified as directly related to chemotherapy treatment (nausea, vomiting, fatigue, and sepsis), with the remaining eight AEs reported to be possibly or probably related. Two subjects each experienced two separate hypokalemic events, with one also reporting nausea and vomiting. One patient who developed sepsis, which was classified as being possibly related to chemotherapy, died as a result of the complication.

## 4. Discussion

LAPC presents significant treatment challenges due to the aggressive nature of the disease, anatomical complexity, and poor prognosis. LAPC often involves critical vasculature, such as the superior mesenteric artery (SMA) and celiac axis. This vascular encasement makes the tumor inoperable in many cases. Surgical resection, the only curative option, becomes impossible or too risky due to the potential for severe bleeding or incomplete tumor removal. While first-line chemotherapy is the preferred management option, LAPC has intrinsic resistance to chemotherapy due to a dense desmoplastic stroma, which limits drug penetration. Common regimens like FOLFIRINOX and gemcitabine-nab-paclitaxel offer limited survival benefits and response rates of approximately 30%, but the patients commonly cannot tolerate more than 3 to 4 months of these regimens. Thus, additional disease control consolidation must be considered [22].

IRE is an emerging treatment for LAPC that offers a non-thermal, less invasive option for tumors deemed inoperable due to vascular involvement. IRE works by delivering high-voltage electrical pulses to create permanent nanopores in the cell membranes, leading to cell death while preserving surrounding structures like blood vessels and ducts. This precision allows it to target tumors near critical anatomy that might not be amenable to traditional surgery or ablation. IRE is typically used in combination with chemotherapy and/or radiation to control local tumor growth. Recent studies suggest that IRE can extend survival and improve local control, but its role remains under limited investigation, with challenges including proper surgeon training, optimal needle placement during the procedure, post-procedural complications, patient selection, and determining optimal timing within the multimodal treatment strategy [9,10,11,12].

The DIRECT Registry study is a prospective, multicenter U.S. IDE real-world study evaluating IRE for the ablative treatment of tumors in patients with Stage 3 PDAC. This preliminary analysis of data from the study demonstrates IRE can be performed safely with low toxicity and favorable 90-day mortality similar to SOC treatment of Stage 3 pancreatic cancer. Because IRE is an invasive treatment and there is a potential for both mild and severe complications, one of the primary objectives of the DIRECT Registry is to focus on the incidence of adverse events in a real-world study population and demonstrate that there is not a significant difference in the incidence of AEs or the severity of AEs when compared to SOC treatment. A recent systematic review summarized the complications reported in studies of the use of IRE therapy for pancreatic cancer [9]. The authors noted that the variability in complication rates observed was likely due to the heterogeneity in treatment protocols and the size of the tumors treated. They also noted that overall experience with IRE therapy may be an additional contributing factor to the complication rate. Tumor size has been reported to be one of the most predictive factors for the development of procedural complications, with larger tumors having the highest complication rates [20]. Subjects enrolled in the DIRECT registry avoided a higher incidence of AEs as a result of strict inclusion criteria both for the biology of the tumor (response to therapy), size of the tumor, and training on the use of IRE for individuals performing the procedure.

The percentage of patients who experienced Grade 3+ AEs in the DIRECT Study was less than what was reported for the multicenter, prospective, single-arm, phase II study of IRE for the treatment of LAPC and isolated local recurrence after pancreatic tumor resection (27.6% vs. 40.0% respectively) [13]. This difference may be a result of the large median tumor diameter for subjects enrolled in the PANFIRE study compared to the DIRECT registry (4.0 vs. 2.2 cm, respectively). The 90-day mortality rate was similar for both studies. Compared to the control group in the present study, IRE treatment was associated with a lower rate of both mild and severe AEs. This is a result of the continued use of chemotherapy, which is the recommendation for LAPC, which has been shown to be associated with a high incidence of overall and Grade 3+ or 4+ AEs [22]. Our results clearly demonstrate that after 3 months of induction chemotherapy, there are substantial chemotherapy-related toxicities with continued use.

The DIRECT registry demonstrates that pausing SOC chemotherapy and then proceeding with definitive, curative intent IRE does not increase the incidence of AEs or the severity. Thus, we do not see a significant effect on the quality of life in these patients during this 90-day recovery after IRE. More importantly, nearly 40% of the patients in the IRE arm had prior radiation, which recent reports have demonstrated increased AEs when performing IRE after radiation [23].

This present analysis of the DIRECT Registry study has several limitations. First, the AE and survival data are limited to a 90-day period following enrollment. Continued follow-up is ongoing until all subjects enrolled reach the 24-month time point. Since it is unlikely that treatment-related AEs would occur beyond this observation period, longer-term safety outcomes associated with the use of IRE would not be anticipated to differ from that currently reported. Additional analyses of longer-term outcomes comparing the IRE and SOC treatment groups, including overall and progression-free survival, are planned. A multivariate analysis will also be conducted to assess the impact of the differing study variables on observed outcomes. There was also an imbalance in the number of patients enrolled in each arm of the study, with three times as many subjects in the IRE treatment arm compared to those treated with SOC. Enrolling patients in a control arm for clinical trials, especially for serious conditions like LAPC, was challenging during the time period for the present study (2019 to 2023). There was patient reluctance since many of the subjects enrolled in the study sought out centers that provided IRE as a treatment. Other patient biases that affected enrollment in the SOC arm included the perception of inferiority of just staying on chemotherapy, the potential toxicity of chemotherapy after 3 months of treatment, and, in some instances, the need for patients to pause chemotherapy. There is also a lack of clear standard of care in LAPC after 3 months of disease control and/or response with induction chemotherapy.

## 5. Conclusions

The initial 90-day results from the DIRECT Registry study provide real-world evidence that in properly selected patients in conjunction with modern SOC chemotherapy, IRE in the treatment of LAPC is safe. Additionally, the use of IRE for curative intent tumor ablation alone or in combination with resection following induction chemotherapy in patients with locally advanced Stage 3 PDAC has acceptable safety and is associated with favorable 90-day mortality in appropriately selected patients. A planned future analysis of the final results from the DIRECT Registry study trial will enable the assessment of longer-term survival, safety, oncological, and quality of life outcomes for subjects enrolled in the trial.

## Figures and Tables

**Table 1:** Patient demographics and clinical characteristics.

	IRE (n = 87)	SOC (n = 27)
Mean age ± SD, years	64.0 ± 8.4	66.4 ± 9.9
Gender, n (%)		
Male	37 (42.5%)	11 (40.7%)
Female	50 (57.5%)	16 (59.3%)
Race, n (%) ^a^		
Asian	0 (0.0%)	2 (7.4%)
Black or African American	5 (5.7%)	4 (14.8%)
White	80 (92.0%)	17 (63.0%)
Ethnicity, n (%) ^a^		
Hispanic or Latino	6 (6.9%)	2 (7.4%)
Not Hispanic or Latino	79 (90.8%)	22 (81.5%)
ECOG performance status		
0	51 (58.6%)	15 (55.6%)
1	36 (41.4%)	11 (40.7%)
Unknown	0 (0.0%)	1 (3.7%)
Median baseline CA19-9, U/mL (Q1–Q3)	29.5 (12.9–86.0) ^b^	112.0 (36.0–761.0)

CA19-9, carbohydrate antigen 19-9; ECOG, Eastern Cooperative Oncology Group; IRE, irreversible electroporation; SD, standard deviation; SOC, standard of care. ^a^: Total percentage does not equal 100% due to missing data. ^b^: n = 79.

**Table 2:** Patient demographics and clinical characteristics.

	IRE (n = 87)	SOC (n = 27)
Tumor location, n (%)		
Head only	47 (54.0%)	12 (44.4%)
Body/neck only	35 (40.2%)	13 (48.1%)
Body/neck; tail	2 (2.3%)	0 (0.0%)
Head; body/neck	3 (3.4%)	2 (7.4%)
Mean anterior/posterior tumor diameter, cm ± SD (number of subjects)	2.2 ± 0.7 (68)	3.2 ± 1.3 (17)
Mean axial tumor diameter, cm ± SD (number of subjects)	2.2 ± 0.7 (68)	2.8 ± 0.8 (17)
Mean cranial-caudal tumor diameter, cm ± SD (number of subjects)	2.4 ± 0.8 (62)	3.0 ± 0.8 (17)
Type of chemotherapy prior to enrollment, n (%) ^a,b^		
FOLFIRINOX	43 (49.4%)	18 (66.7%)
FOLFIRINOX + Gemcitabine	5 (5.7%)	0 (0.0%)
FOLFIRINOX + Gemcitabine/Abraxane	19 (21.8%)	1 (3.7%)
FOLFOX	1 (1.1%)	0 (0.0%)
Gemcitabine/Abraxane	15 (17.2%)	6 (22.2%)
Other combination	1 (1.1%)	1 (3.7%)
Mean chemotherapy cycles prior to enrollment, n ± SD	5.7 ± 5.0	4.6 ± 2.8
Prior radiation therapy, n (%) ^a^		
Yes	37 (42.5%)	0 (0.0%)
No	44 (50.6%)	25 (92.6%)

IRE, irreversible electroporation; SD, standard deviation; SOC, standard of care. ^a^: Total percentage does not equal 100% due to missing data. ^b^: n = 79.

**Table 3:** Procedural details for IRE subjects (n = 87).

Mean Time from Diagnosis to IRE, Months ± SD	9.2 ± 4.9
Approach, n (%)	
Open	87 (100%)
Probe configuration, n (%) ^a^	
Diamond	10 (11.5%)
Line	24 (27.6%)
Square	8 (9.2%)
Three Triangles	3 (3.4%)
Triangle	15 (17.2%)
Multiple	4 (4.6%)
Electrode exposure (cm), n (%) ^a^	
1.0	1 (1.1%)
1.0–1.5	2 (2.3%)
1.5	32 (36.8%)
2.0	3 (3.4%)
Mean number of pulses delivered (n = 65), n ± SD	1031.1 ± 882.1
Pulse length (μs), n (%) ^a^	
70	1 (1.1%)
70–90	8 (9.2%)
80–90	3 (3.4%)
90	54 (62.1%)
Mean total IRE delivery time (n = 66), minutes ± SD	55.7 ± 54.5
Mean procedure time, minutes ± SD	338.0 ± 174.2
Pullback required, n (%) ^a^	
Yes	35 (40.2%)
No	29 (33.3%)
Number of pullbacks (n = 35), n ± SD	2.0 ± 1.1
High current events, n (%) ^a^	
Yes	24 (27.6%)
No	42 (48.3%)
Low voltage events, n (%) ^a,b^	
Yes	13 (14.9%)
No	53 (60.9%)
Pancreatic resections	
Whipple	25 (28.7%)
Distal pancreatectomy	5 (5.7%)
Subtotal pancreatectomy	3 (3.4%)
Subtotal pancreatectomy with celiac resection	2 (2.3%)
Adjunctive procedures ^c^	
Cholecystectomy	29 (33.3%)
J-tube	25 (28.7%)
Lymphadenectomy	13 (14.9%)
Splenectomy	8 (9.2%)
Hepaticojejunostomy	8 (9.2%)
Gastrojejunostomy	6 (6.9%)
Bile duct resection	5 (5.7%) ^c^
Portal vein or SMV resection	4 (4.6%)
Bile duct resection	4 (4.6%)
Bile duct resection and pyloric exclusion	2 (2.3%)
Other	13 (14.9%) ^d^

^a^: Total percentage does not equal 100% due to missing data. ^b^: Low voltage events occur when the NanoKnife system delivers less than 1500 volts/cm. ^c^: Includes one subject who also had a metal stent removed. ^d^: Adjunctive procedures performed in a single subject are listed in Supplemental Table S3.

**Table 4:** CTCAE Grade 3 or greater adverse events from enrollment to day 90.

Body System	Dictionary Term	IRE Treatment ^a^ (n = 87)	Standard of Care (n = 27) ^b^
		Subjects, n (%)	Subjects, n (%)
Overall		24 (27.6%)	12 (44.4%)
Blood And lymphatic system disorders		6 (6.9%)	4 (14.8%)
	Anemia	6 (6.9%)	3 (11.1%)
	Neutropenia	0 (0.0%)	2 (7.4%)
	Thrombocytopenia	1 (1.1%)	0 (0.0%)
Cardiac disorders		4 (4.6%)	1 (3.7%)
	Atrial fibrillation	1 (1.1%)	1 (3.7%)
	Cardiac arrest	1 (1.1%)	0 (0.0%)
	Cardiopulmonary arrest	1 (1.1%)	0 (0.0%)
	Cardiorespiratory distress	1 (1.1%)	0 (0.0%)
	Ventricular arrhythmia	1 (1.1%)	0 (0.0%)
Gastrointestinal disorders		11 (12.6%)	5 (18.5%)
	Abdominal pain	3 (3.4%)	1 (3.7%)
	Ascites	1 (1.1%)	0 (0.0%)
	Constipation	0 (0.0%)	1 (3.7%)
	Duodenal obstruction	1 (1.1%)	0 (0.0%)
	Gastrointestinal hemorrhage	3 (3.4%)	0 (0.0%)
	Hematemesis	1 (1.1%)	0 (0.0%)
	Intraabdominal hemorrhage	1 (1.1%)	0 (0.0%)
	Nausea	0 (0.0%)	1 (3.7%)
	Pancreatic failure	0 (0.0%)	1 (3.7%)
	Pancreatitis	2 (2.3%)	0 (0.0%)
	Pneumatosis intestinalis	1 (1.1%)	0 (0.0%)
General disorders and administration site conditions		3 (3.4%)	4 (14.8%)
	Adverse drug reaction	0 (0.0%)	1 (3.7%)
	Asthenia	1 (1.1%)	0 (0.0%)
	Fatigue	0 (0.0%)	1 (3.7%)
	Malaise	0 (0.0%)	1 (3.7%)
	Pain	1 (1.1%)	1 (3.7%)
	Pyrexia	1 (1.1%)	0 (0.0%)
Hepatobiliary disorders		2 (2.3%)	1 (3.7%)
	Bile duct obstruction	1 (1.1%)	0 (0.0%)
	Biliary tract disorder	1 (1.1%)	0 (0.0%)
	Cholangitis	1 (1.1%)	0 (0.0%)
	Hyperbilirubinemia	0 (0.0%)	1 (3.7%)
Infections and infestations		16 (18.4%)	5 (18.5%)
	Abdominal abscess	2 (2.3%)	0 (0.0%)
	Abdominal infection	2 (2.3%)	0 (0.0%)
	Arthritis bacterial bacteremia	0 (0.0%)	1 (3.7%)
		1 (1.1%)	0 (0.0%)
	Biliary tract infection bacterial	1 (1.1%)	0 (0.0%)
	Clostridiodes difficile sepsis	1 (1.1%)	0 (0.0%)
	Clostridium difficile infection	1 (1.1%)	0 (0.0%)
	Emphysematous cholecystitis	0 (0.0%)	1 (3.7%)
	Gastroenteritis	1 (1.1%)	0 (0.0%)
	Hepatic infection	1 (1.1%)	0 (0.0%)
	Pancreatic abscess	1 (1.1%)	0 (0.0%)
	Pneumonia	1 (1.1%)	0 (0.0%)
	Postoperative wound infection	1 (1.1%)	0 (0.0%)
	Retroperitoneal abscess	1 (1.1%)	0 (0.0%)
	Sepsis	5 (5.7%)	1 (3.7%)
	Septic shock	3 (3.4%)	0 (0.0%)
	Urinary tract infection	0 (0.0%)	1 (3.7%)
	Vascular access site infection	1 (1.1%)	0 (0.0%)
Injury, poisoning and procedural complications		4 (4.6%)	1 (3.7%)
	Lower limb fracture	0 (0.0%)	1 (3.7%)
	Post-procedural bile leak	1 (1.1%)	0 (0.0%)
	Ureteric injury	1 (1.1%)	0 (0.0%)
	Vascular pseudoaneurysm	2 (2.3%)	0 (0.0%)
Investigations		0 (0.0%)	2 (7.4%)
	Neutrophil count decreased	0 (0.0%)	1 (3.7%)
	Weight decreased	0 (0.0%)	1 (3.7%)
Metabolism and nutrition disorders		1 (1.1%)	5 (18.5%)
	Decreased appetite	0 (0.0%)	1 (3.7%)
	Dehydration	0 (0.0%)	2 (7.4%)
	Hypoalbuminemia	1 (1.1%)	0 (0.0%)
	Hypokalemia	0 (0.0%)	3 (11.1%)
Musculoskeletal and connective tissue disorders		0 (0.0%)	1 (3.7%)
	Back pain	0 (0.0%)	1 (3.7%)
Nervous system disorders		1 (1.1%)	2 (7.4%)
	Syncope	1 (1.1%)	2 (7.4%)
Product issues		0 (0.0%)	1 (3.7%)
	Device occlusion	0 (0.0%)	1 (3.7%)
Psychiatric disorders		2 (2.3%)	1 (3.7%)
	Confusional state	1 (1.1%)	0 (0.0%)
	Delirium	1 (1.1%)	0 (0.0%)
	Depression	0 (0.0%)	1 (3.7%)
Renal and urinary disorders		1 (1.1%)	0 (0.0%)
	Acute kidney injury	1 (1.1%)	0 (0.0%)
Respiratory, thoracic, and mediastinal disorders		3 (3.4%)	0 (0.0%)
	Pleural effusion	1 (1.1%)	0 (0.0%)
	Respiratory failure	2 (2.3%)	0 (0.0%)
Surgical and medical procedures		1 (1.1%)	0 (0.0%)
	Hepatic embolization	1 (1.1%)	0 (0.0%)
Vascular disorders		4 (4.6%)	0 (0.0%)
	Arterial hemorrhage	1 (1.1%)	0 (0.0%)
	Hypotension	1 (1.1%)	0 (0.0%)
	Pseudoaneurysm	1 (1.1%)	0 (0.0%)
	Shock hemorrhagic	1 (1.1%)	0 (0.0%)

CTCAE, Common Terminology Criteria for Adverse Events; IRE, irreversible electroporation. ^a^: IRE treatment-related Grade 4+ events from treatment to study Day 90. ^b^: Chemotherapy-related Grade 4+ events from enrollment to study Day 90.

**Table 5:** CTCAE Grade 4 or greater adverse events from enrollment to day 90.

System Organ Class	Preferred Term	IRE Treatment ^a^ (n = 87)		Standard of Care ^b^ (n = 27)	
		Subjects, n (%)	Events	Subjects, n (%)	Events
Any Grade 4+ adverse event		3 (3.4%)	4	7 (25.9%)	12
Metabolism and nutrition disorders	All	0 (0.0%)	0	2 (7.4%)	4
	Hypokalemia	0 (0.0%)	0	2 (7.4%)	4
Infections and infestations	All	1 (1.1%)	1	4 (14.8%)	4
	Sepsis	0 (0.0%)	0	2 (7.4%)	2 ^c^
	Septic Shock	1 (1.1%)	1	0 (0.0%)	0
	Bacteremia	0 (0.0%)	0	1 (3.7%)	1 ^d^
	Urinary tract infection	0 (0.0%)	0	1 (3.7%)	1
Gastrointestinal disorders	All	0 (0.0%)	0	1 (3.7%)	2
	Nausea	0 (0.0%)	0	1 (3.7%)	1
	Vomiting	0 (0.0%)	0	1 (3.7%)	1
Product issues	All	0 (0.0%)	0	1 (3.7%)	1
	Device occlusion	0 (0.0%)	0	1 (3.7%)	1 ^e^
General disorders and administration site conditions	All	0 (0.0%)	0	1 (3.7%)	1
	Fatigue	0 (0.0%)	0	(3.7%)	1
Vascular disorders	All	1 (1.1%)	1	0 (0.0%)	0
	Arterial Hemorrhage/abdominal bleed	1 (1.1%)	1 ^f^	0 (0.0%)	0
Injury, poisoning, and procedural complications	All	1 (1.1%)	1	0 (0.0%)	0
	Vascular pseudoaneurysm	1 (1.1%)	1 ^f^	0 (0.0%)	0
Cardiac disorders	All	1 (1.1%)	1	0 (0.0%)	0
	Cardiac arrest	1 (1.1%)	1	0 (0.0%)	0

^a^: IRE treatment-related Grade 4+ events from treatment to study Day 90. ^b^: Chemotherapy-related Grade 4+ events from enrollment to study Day 90. ^c^: event resulted in death for one subject. ^d^: *E. coli* bacteremia with associated septic shock. ^e^: occluded stent. ^f^: outcome was subject death.

## Data Availability

The datasets presented in this article are not readily available because the data are part of an ongoing study. Data are planned to be shared upon the completion of the study and its submission to the FDA. The data supporting this study’s findings will be available from the corresponding author upon reasonable request.

## Supplementary Materials

The following supporting information can be downloaded at: https://www.mdpi.com/article/10.3390/cancers16233894/s1, Table S1: Prior medical history at the time of enrollment; Table S2: Pre- and post-enrollment surgical history; Table S3: Adjunctive procedures performed in a single subject.

## Clinical Trial Registration

ClinicalTrials.gov identifier: NCT03899649.
