# Supplementary material for: Effectiveness and Safety of Irreversible Electroporation When Used for the Ablation of Stage 3 Pancreatic Adenocarcinoma: Initial Results from the DIRECT Registry Study
Source: Cancers (Basel). 2024 Nov 21;16(23):3894. doi: 10.3390/cancers16233894 (PMC11640091; doi:10.3390/cancers16233894)
Supplement: Supplementary file 1 [file cancers-16-03894-s001.zip › cancers-3285617-supplementary.pdf]

Supplementary Materials

Effectiveness and Safety of Irreversible Electroporation When Used for the Ablation of Stage 3 Pancreatic Adenocarcinoma: Initial Results from the DIRECT Registry Study

Table S1. Prior Medical History at the Time of Enrollment

Category	IRE (n=87)	SOC (n=27)
Blood and lymphatic system disorders	14 (16.1%)	7 (25.9%)
Cardiac disorders	16 (18.4%)	4 (14.8%)
Congenital, familial and genetic disorders	1 (1.1%)	1 (3.7%)
Ear and labyrinth disorders	4 (4.6%)	0 (0.0%)
Endocrine disorders	11 (12.6%)	1 (3.7%)
Eye disorders	4 (4.6%)	1 (3.7%)
Gastrointestinal disorders	44 (50.6%)	10 (37.0%)
General disorders and administration site conditions	4 (4.6%)	1 (3.7%)
Hepatobiliary disorders	5 (5.7%)	3 (11.1%)
Immune system disorders	4 (4.6%)	0 (0.0%)
Infections and infestations	7 (8.0%)	2 (7.4%)
Injury, poisoning and procedural complications	4 (4.6%)	1 (3.7%)
Investigations	7 (8.0%)	3 (11.1%)
Metabolism and nutrition disorders	42 (48.3%)	17 (63.0%)
Musculoskeletal and connective tissue disorders	11 (12.6%)	6 (22.2%)
Neoplasms benign, malignant and unspecified (including cysts and polyps)	21 (24.1%)	3 (11.1%)
Nervous system disorders	23 (26.4%)	3 (11.1%)

Psychiatric disorders	13 (14.9%)	7 (25.9%)
Renal and urinary disorders	10 (11.5%)	4 (14.8%)
Reproductive system and breast disorders	7 (8.0%)	3 (11.1%)
Respiratory, thoracic and mediastinal disorders	22 (25.3%)	4 (14.8%)
Skin and subcutaneous tissue disorders	6 (6.9%)	0 (0.0%)
Surgical and medical procedures	6 (6.9%)	2 (7.4%)
Vascular disorders	47 (54.0%)	10 (37.0%)

IRE, Irreversible electroporation; SOC, Standard of care

Table S2. Pre- and Post Enrollment Surgical History

	IRE Treatment* (n=87)		Standard of Care* (n=27)	
Category	Prior to Enrollment	Post Enrollment**	Prior to Enrollment	Post Enrollment
Appendectomy	8 (9.2%)	0 (0.0%)	1 (3.7%)	0 (0.0%)
Biliary Bypass	0 (0.0%)	1 (1.1%)	1 (3.7%)	0 (0.0%)
Breast	3 (3.4%)	1 (1.1%)	5 (18.5%)	1 (3.7%)
Cardiac	7 (8.0%)	0 (0.0%)	2 (7.4%)	0 (0.0%)
Cholecystectomy	13 (14.9%)	1 (1.1%)	4 (14.8%)	0 (0.0%)
Colectomy	3 (3.4%)	0 (0.0%)	0 (0.0%)	0 (0.0%)
Endoscopy	7 (8.0%)	1 (1.1%)	2 (7.4%)	0 (0.0%)
Exploratory Laparotomy	5 (5.7%)	2 (2.3%)	1 (3.7%)	2 (7.4%)
Gastric	7 (8.0%)	0 (0.0%)	1 (3.7%)	1 (3.7%)
Obstetrics/Gynecology	17 (19.5%)	0 (0.0%)	6 (22.2%)	0 (0.0%)
Orthopedic	2 (2.3%)	0 (0.0%)	4 (14.8%)	0 (0.0%)
Pancreatectomy	4 (4.6%)	2 (2.3%)	0 (0.0%)	4 (14.8%)
Paracentesis	0 (0.0%)	1 (1.1%)	0 (0.0%)	0 (0.0%)
Prostate	1 (1.1%)	1 (1.1%)	1 (3.7%)	0 (0.0%)
Small Bowel Resection	0 (0.0%)	0 (0.0%)	1 (3.7%)	0 (0.0%)
Spine	1 (1.1%)	0 (0.0%)	0 (0.0%)	0 (0.0%)
Splenectomy	0 (0.0%)	0 (0.0%)	0 (0.0%)	3 (11.1%)
Vascular	1 (1.1%)	1 (1.1%)	0 (0.0%)	1 (3.7%)

*Reported as number of subjects (percent of subjects in study arm)

** Excludes procedures performed as adjunctive procedures to IRE treatment.

IRE, Irreversible electroporation

Table S3. Adjunctive Procedures Performed in a Single Subject

Adjunctive Procedure
Celiac axis resection
Duodenal-jejunal resection
Duodenojejunostomy and partial duodenectomy
Exploratory laparotomy, partial duodenal exclusion, Roux-en-Y duodenojejunostomy, and diagnostic laparoscopy
Major vascular reconstruction of portal vein and partial right hepatic lobectomy
Omentectomy
Omentectomy and partial left hepatic lobectomy
Omentum flap and open abdominal cavity lymph node excision
Open side-to-side choledochoduodenostomy biliary bypass and removal of common bile duct stent
Partial right haptic lobectomy and intra-abdominal lymphadenectomy, proximal jejunum and 4th duodenum with Roux-en-Y reconstruction
Pylorus-preserving pancreaticoduodenectomy
Pylorus-preserving pancreaticoduodenectomy and omentectomy.
